# Flammability, Smoke, Mechanical Behaviours and Morphology of Flame Retarded Natural Fibre/Elium^®^ Composite

**DOI:** 10.3390/ma12172648

**Published:** 2019-08-21

**Authors:** Pooria Khalili, Brina Blinzler, Roland Kádár, Roeland Bisschop, Michael Försth, Per Blomqvist

**Affiliations:** 1 Material and Computational Mechanics, Department of Industrial and Materials Science, Chalmers University of Technology, 412 96 Gothenburg, Sweden; 2 Division of Engineering Materials, Department of Industrial and Materials Science, Chalmers University of Technology, 412 96 Gothenburg, Sweden; 3 Division Safety and Transport/Safety/Fire Research, RISE Research Institutes of Sweden, 501 15 Borås, Sweden; 4 Division Structural and Fire Engineering, Department of Civil, Environmental and Natural Resources Engineering, Luleå University of Technology, 97 187 Luleå, Sweden

**Keywords:** polymer-matrix composites, mechanical properties, Elium^®^

## Abstract

The work involves fabrication of natural fibre/Elium^®^ composites using resin infusion technique. The jute fabrics were treated using phosphorus-carbon based flame retardant (FR) agent, a phosphonate solution and graphene nano-platelet (GnP), followed by resin infusion, to produce FR and graphene-based composites. The properties of these composites were compared with those of the Control (jute fabric/Elium^®^). As obtained from the cone calorimeter and Fourier transform infrared spectroscopy, the peak heat release rate reduced significantly after the FR and GnP treatments of fabrics whereas total smoke release and quantity of carbon monoxide increased with the incorporation of FR. The addition of GnP had almost no effect on carbon monoxide and carbon dioxide yield. Dynamic mechanical analysis demonstrated that coating jute fabrics with GnP particles led to an enhanced glass transition temperature by 14%. Scanning electron microscopy showed fibre pull-out locations in the tensile fracture surface of the laminates after incorporation of both fillers, which resulted in reduced tensile properties.

## 1. Introduction

The automotive industry and aerospace manufacturers prefer composites of biodegradable or/and renewable materials. This sort of development is ongoing and the replacement of synthetic fabrics with natural fabrics (NFs) brings prominent benefits, for example, lower cost, lower density, high specific strength and lower weight [1,2,3,4,5]. This would even be better if the composite is recyclable or/and biodegradable. Amongst all NFs, kenaf, sisal, flax, jute, cotton, hemp and ramie are known to be the most widely used lignocellulosic fibres.

**Figure 1:**
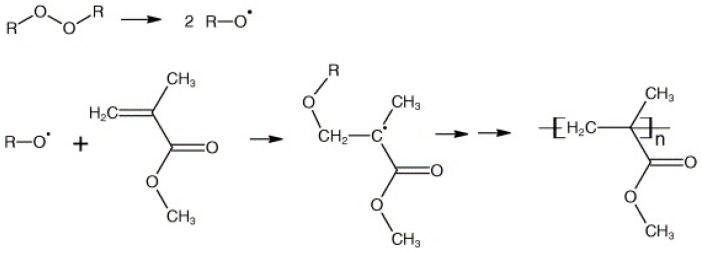
Free radical vinyl polymerization of methyl methacrylate (MMA) to poly methyl methacrylate (PMMA) with dibenzoyl peroxide initiator [11,12].

Thermoset resins are widely used in industry due to their average curing temperature and improved mechanical performances. However, thermoplastic resins need to be processed at elevated temperatures, and therefore, they are used for specific applications in packaging, construction and automotive industries. In particular, when it comes to the usage of NFs in composite this topic is of prominent importance as they degrade at high temperatures. E.g., poly-lactic acid (PLA), polypropylene (PP), cyclic butylene terephthalate (CBT), polyurethanes (PU), polyamide 6 (PA6) and many more need high processing temperatures [5,6,7]. However, thermoplastics have appealing properties such as recyclability [8], high impact performance [8], post formability [9] and good vibration dampening capacities [10]. Exceptionally, Elium^®^ thermoplastic can be processed at room temperature. It undergoes free radical polymerisation from methyl methacrylate (MMA), i.e., its monomer, to poly methyl methacrylate (PMMA), i.e., its polymer with peroxide initiators [11,12] (Figure 1). The initiator is a dibenzoyl peroxide in the form of white free flowing powder with 50% dicyclohexyl phthalate and the major decomposition products, according to the manufacturer, include carbon dioxide, benzene, benzoic acid, diphenyl and phenylbenzoate. ARKEMA highlights the merit of this resin as being weldable, recyclable and superior in fatigue, impact performance and part manufacturing costs lesser than other thermoplastic technologies.

Natural fibre/Elium^®^ composite laminates have tremendous potential to be used for secondary composite applications due to their combined mechanical and dampening properties. These composite systems can be produced using resin infusion methods at ambient temperature, and this process is currently used by numerous industries such as aerospace, rail, marine sports and light rail [13]. The technique is cost effective and can produce large and complex components with relatively good mechanical performances. 

The main challenge in the usage of natural fibre composites is their flammability [14], and this limits their usage in applications where the fire safety regulations are stringent such as marine, aviation and railway industries [2]. The heat release from NF composites are higher than that of their carbon and glass fibre counterparts [15]. Therefore, flame retardants are required to be used in order to reduce the heat release and flammability of NF composites. In this work, to prohibit the filtration of flame retardant (FR) particles with the tubing systems (spiral, PVC tubes) and also the fabric itself during resin infusion, the FR additives were not mixed with the liquid resin but instead NFs were flame retarded by their impregnation in FR solutions, followed by a pad-dry cure prior to the infusion. It is one of the widely used textile finishing processes for coating the fabrics and fibres with FRs. Several phosphorous based inorganic compounds, i.e., ammonium phosphates and phosphoric acid, were applied onto the natural fibres to improve the flame retardancy of thermoplastic composites [16,17,18]. The results demonstrated a trade-off between the composite strength and heat release properties.

To the best of authors’ knowledge, very few works have been reported the combination of both jute fabrics and a recyclable acrylic based resin to make NF reinforced composites and to characterise them. The fabric reinforced thermoplastic composites were fabricated using resin infusion technique, which is a very common method for producing the thermoset composites, as the resin is in the liquid form. The composite laminates were processed at only room temperature unlike other thermoplastic counterparts which require high processing temperatures that lead to the thermal degradation of NFs, and energy saving can be improved significantly. Besides, the effect of treating natural fabrics using an environmentally friendly, biodegradable FR agent and GnP particles on the heat release properties, smoke, viscoelastic behaviour, mechanical performance and morphology of the jute fabric Elium^®^ based composite laminates were investigated for the first time.

## 2. Materials and Methods

### 2.1. Materials

**Figure 2:**
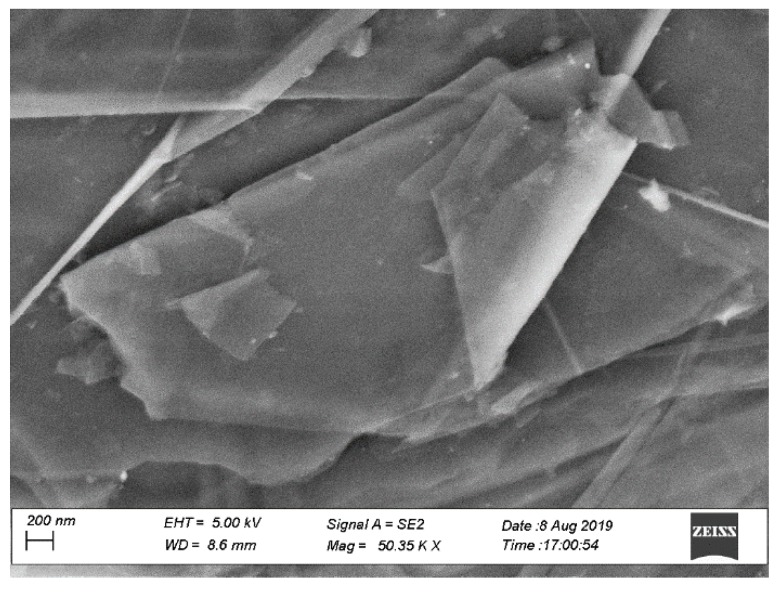
Scanning electron microscope of graphene nano-platelet (GnP) powder.

Untreated plain-woven jute fabrics were purchased from Stoff & Stil (Gothenburg, Sweden) and had the surface mass of 402 g/m^2^. A two-component Elium^®^ resin system (Elium^®^ 150) was provided by the company Arkema (Colombes, France). Dibenzoyl peroxide was used as a hardener for the acrylic (Elium^®^) resin. The resin had the viscosity of 100 mPa·s and the liquid density was 1.01 g/cm^3^. An aqueous organic phosphonate ester (Exfire T) flame-retardant (FR) agent (part number: 18-9100-30) was supplied by Dafo Brand AB company (Tyresö, Sweden). In order to shed light on the chemical structure of the flame retardant (FR), the infrared spectroscopy (IR) was carried out (not shown here) and revealed the characteristic peaks of organophosphate-based FR together with N–H bands. The latter bands are related to the alkaline ammonia, which was added to FR in order to reduce its pH of acidic organic phosphonate ester. It should be noted that the FR is a halogen-free compound, which is environmentally friendly. It is biodegradable, and possesses neutral pH as received. According to the supplier, it does not cause allergic reactions or any other health hazards in normal handling. The product has 27 wt.% solid content, and the quantity of carbon, nitrogen and phosphorus elements were 3.6 wt.%, 14 wt.% and 12 wt.% in the solid content, respectively. Graphene nano-platelets grade M (GnP) was purchased from XG Sciences Inc. (Lansing, MI, USA). GnP particles consist of short stacks of graphene sheets forming a platelet shape. The platelets had a surface area of 120–150 m^2^/g, thickness of 6–8 nm, average diameter of 5 μm and density of 2.2 g/cm^3^, according to the manufacturer (Figure 2).

### 2.2. Fibre Treatment

**Figure 3:**
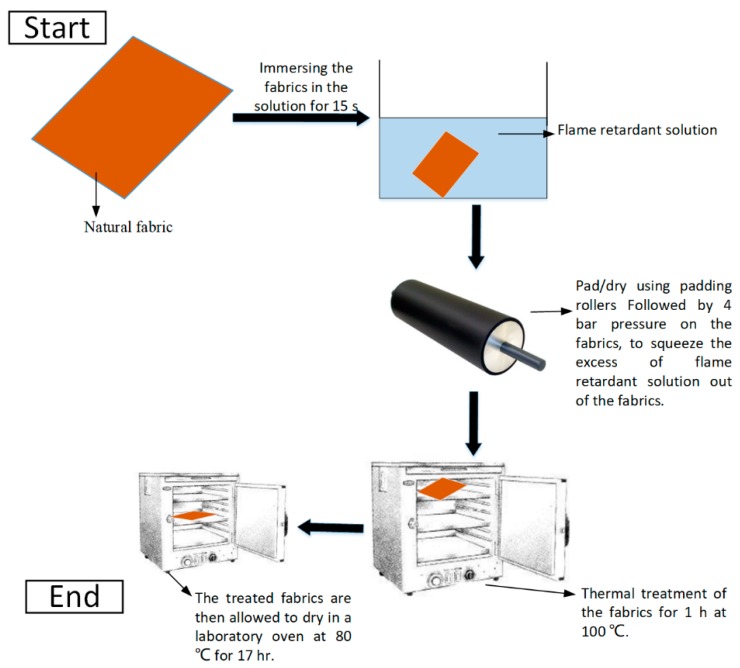
Schematic illustration of fibre treatment.

For the flame-retardant treatment of jute fabrics, one-part aqueous FR and one-part water were mixed manually for 2 min and the fabrics were soaked in the solution for 15 s. The fabrics were pad/dried using padding rollers and then pressed at 4 bars using a manual cold press (50T Bahco hydraulic press, Rotherham, UK) to squeeze the excess of FR solution out of the fabrics. The fabrics were kept in the oven for an hour (thermal treatment to allow a good degree of adhesion between the fabric and additives) at 100 °C to improve the adhesion between the fibres and particles, and subsequently, they were dried for 17 h at 80 °C prior to the composite fabrication (Figure 3). The mass uptake was measured for each layer of fabric. The adjustments (the fabric soaking time and the ratio of FR to water) were made to incorporate 6 wt.% of FR additives in its respective composite. This means the total amount of phosphorus and carbon in the final composite is about 1 wt.% at the weight ratio of 3.3:1 (P/C).

For the GnP treatment of fabrics, the solution of GnP and water (1 g to 100 mL, respectively) was prepared using a Bransonic 1510E-MT ultrasonication (Danbury, CT, USA) at the frequency 42 kHz for 2 h. The fabrics were soaked for 15 s in the GnP solution, were kept for an hour at 100 °C in a laboratory oven, and then were dried for 17 h at 80 °C prior to the composite fabrication. The amount of GnP coated onto the surface of fabrics was measured. This fabric soaking time and the GnP:water ratio in the solution were used to obtain 1 wt.% GnP in jute fabric Elium^®^ composite. Higher concentration of GnP filler on the fabrics led to an obvious agglomeration of fillers on the jute fabric, which is why this specific concentration was used as the maximum quantity of GnP additives without visual sign of aggregates.

### 2.3. Resin Infusion

The processing started with applying the gum tape around the perimeter of the mould. Then, two layers of dry jute fabrics were placed onto a steel mould, which was already surface coated with a layer of PTFE release agent followed by another layer of PVA release agent. A layer of peel ply was positioned on the fabrics, and subsequently an infusion spiral and two silicon connectors were placed on the peel ply (one near inlet and the other one near outlet). The flash tape was used to stick the parts in place. The vacuum bagging film was adhered to the gum tape and the resin feed PVC hose and outlet PVC hose were connected to the system through the silicon connectors. A line clamp was utilised to close the inlet PVC tube and evacuate the air from the set-up by the vacuum pump. The whole system was released from the vacuum and vacuum was applied again three subsequent times to compact the fabrics, which led to a consistent fibre volume fraction. After reaching the desired vacuum, the sealed system was left for 10 min to ensure the absence of leakage. 

**Figure 4:**
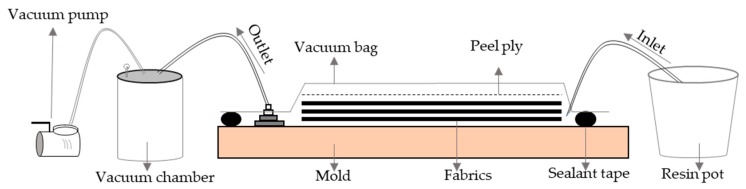
Graphical illustration of resin infusion method.

A stochiometric ratio of dibenzoyl peroxide was added to the Elium^®^ at the weight ratio of 1.5 to 100, respectively, and stirred manually for 2 min. The mixture was resin infused onto the fabric in the mould (Figure 4). The part was left to cure for 24 h at the ambient temperature. Subsequently, the composites were demoulded and cut into specified sample dimensions using diamond saw machine for different tests. The fibre volume fraction was 40% for the composites measuring the thickness of 1.5 mm (Table 1). The composite with the presence of only natural fabric and polymer was designated as the Control, and the composites containing 0.72 wt.% P together with 0.22 wt.% C and 1 wt.% GnP nominated FR composite and GnP composite, respectively.

### 2.4. Cone Calorimeter Test

The fire behaviour of the composite laminates was investigated using cone calorimetry (Fire Testing Technology). Square samples measuring 100 mm × 100 mm were tested at a constant heat flux of 35 kW/m^2^ in accordance with ISO 5660-1. Time to ignition (TTI), which is the time it takes for the specimen to ignite after being exposed to the heat source, peak heat release rate (PHRR) which is the peak of heat release curve during the period of combustion indicating the maximum intensity of a fire, time to PHRR (t_PHRR_) which is the time it takes for the specimen to reach its peak heat release rate, total heat release (THR) which refers to the time integral of heat release rate curve during the whole process of burning, the effective heat of combustion (EHC) which denotes the amount of heat generated by burning a unit quantity of a material, specific extinction area (SEA), which demonstrates the quantity of smoke emitted per unit of mass of volatile generated upon heating, rate of smoke release (RSR) and total smoke release (TSR), which is calculated by the integration of RSR curve, were obtained. The fire growth index (FIGRA), which is to qualify the properties of fire reactions of materials, was also calculated, based on the ratio of PHHR to t_PHRR_.

### 2.5. Fourier Transform Infrared Spectrometer (FTIR) Test

The cone calorimeter was connected to a Fourier transform infrared spectroscopy (FTIR) instrument to measure the amount of carbon monoxide (CO) and carbon dioxide (CO_2_) in accordance with ISO 19702:2015. The FTIR spectrometer (Thermo Scientific Antaris IGS analyze, Waltham, MA, USA) had a resolution of 0.5 cm^−1^, spectral range of 4800–650 cm^−1^, scans over spectrum of 10, and time per spectrum of 12 s.

### 2.6. Dynamic Mechanical Analysis

Dynamic mechanical analysis (DMA) tests were carried out in three-point bending mode on a Rheometrics Solids Analyzer RSA II (TA Instruments, New Castle, DE, USA). The samples used had the dimension of 50 mm × 10 mm × 1.5 mm. Temperature was ramped from 30 °C to 170 °C at a constant rate of 5 °C/min. The frequency was set to 1 Hz, and the storage modulus and loss factor (tan δ) were obtained. The strain amplitude was opted based on the strain sweep test to be in the linear viscoelastic region. The strain amplitude obtained was set to 0.004 m/m.

### 2.7. Scanning Electron Microscopy

Scanning electron microscopy (SEM) micrographs of the fracture surfaces of tensile samples were obtained using an environmental scanning microscope Philips XL-30 field emission ESEM (Amsterdam, Netherlands) with an accelerating voltage of 5 kV. The samples were gold sputtered using a sputter coater Edwards S150B (Perth, UK) in vacuum with 60 s plasma exposition prior to the scanning.

### 2.8. Tensile Test

The tensile properties of the composite laminates were evaluated using a Bent Tram A/S test machine (Aalborg, Denmark) in accordance with BS EN ISO 527. Four rectangular specimens of the dimension of 150 mm × 20 mm × 1.5 mm were characterised for each composite. To ensure failure within the gauge region, 25 mm long tabs were attached to the ends of the samples, and the gauge length was 100 mm. The machine had the load-cell of 50 kN and the cross-head speed was set 1 mm/min. The stress-strain curves were obtained, and the tensile parameters were measured. 

### 2.9. Flexural Test

The flexural (three-point bending) behaviours of the composite laminates were studied using a Bent Tram A/S test machine (the same instrument used for the tensile test) in accordance with BS EN ISO 14125. Four rectangular specimens measuring 60 mm ×15 mm × 2 mm with the span length of 40 mm were tested for each composite. The cross-head speed was set to 1 mm/min and the instrument load-cell was 50 kN. The stress-deformation curves were obtained, and the flexural parameters were recorded.

## 3. Results

### 3.1. Flammability and Smoke Properties

**Figure 5:**
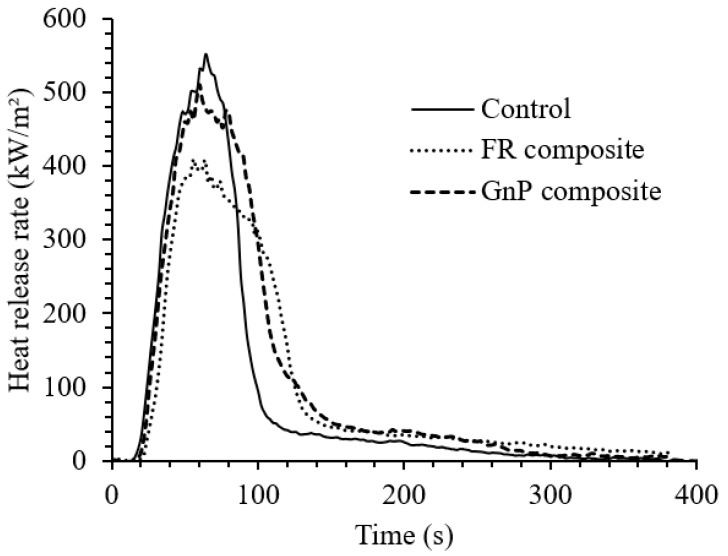
Heat release rate (HRR) of the composites.

**Figure 6:**
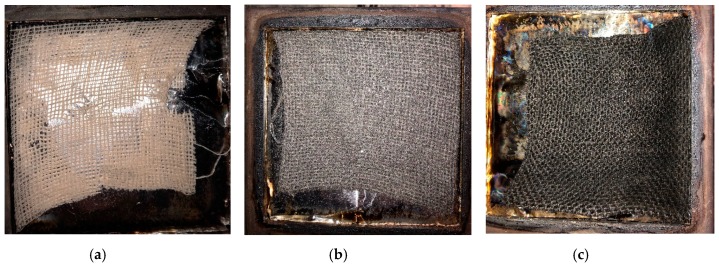
The char residue of (**a**) the Control, (**b**) GnP composite and (**c**) FR composite after the cone calorimeter test.

**Figure 7:**
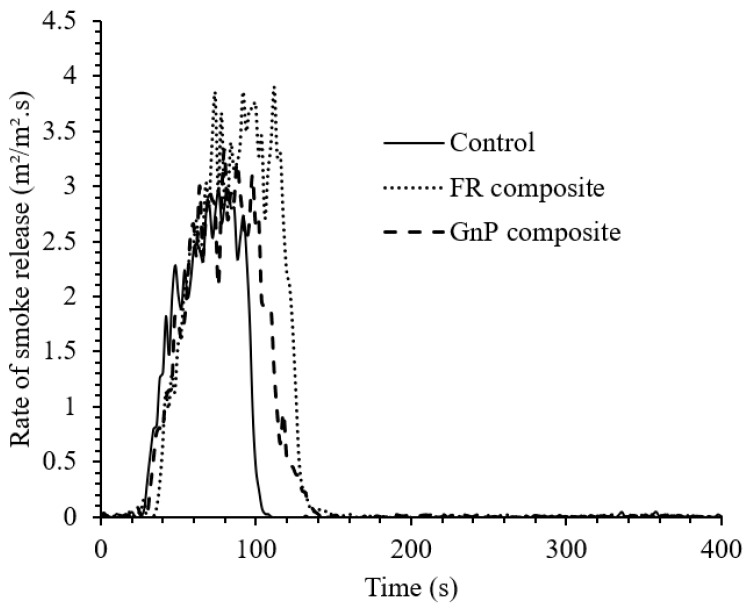
Rate of smoke release (RSR) results of the composites.

The flammability of jute Elium^®^ composites was evaluated using the cone calorimeter method and a connected FTIR instrument for gas analysis. The cone calorimeter results of the Control, GnP composite and FR composite are shown in Figure 5, Figure 6 and Figure 7, Table 2 and Table 3.

The jute/Elium^®^ composite (Control) ignited at 21 s and yielded a peak heat release rate (PHRR) of 552 kW/m^2^. The Control produced a total heat release (THR) of 33.5 MJ/m^2^, resulting in an effective heat of combustion (EHC) of 20.6 MJ/kg. All these data points refer to the flaming phase of the cone calorimeter test. With the addition of FR and GnP fillers into the composites, the time to ignition (TTI) was prolonged and the PHRR and FIGRA reduced. The decrease in PHRR was more significant for FR composite by 25% reduction relative to that of the Control. This is attributed to the formation of more char residue after the inclusion of the FR, which contains carbon, phosphorus and nitrogen elements, and GnP that contains carbon. The char acts as a thermal barrier on the composite surface to shield the underlying materials from the fire zone and heat, as demonstrated by the residue (Figure 6b,c). No indication of char residue was seen on the Control sample (Figure 6a). The Control burned off and was torn, and a small amount of fabric left as the residue. The FR and GnP composite shrunk slightly but preserved the structure of the fabrics (Figure 6b,c). This formation of char demonstrates the influence of the fillers in the condensed phase. While the specimens were burning, it was observed that the Elium^®^ polymer first melted, then decomposed by end chain scission with the loss of individual monomer units and eventually evaporated. However, the t_PHRR_ reduced slightly, and the THR increased a little after addition of the FR and GnP additives since the filled composites burned for slightly longer time; Branda et al. [19] reported that treating the hemp fabrics with water-glass slightly increase the THR of hemp fabric epoxy composite. The addition of fillers in the composites had very little influence on the EHC results. Between the FR and GnP composites, it was observed that incorporation of FR additives slowed down the intensity of flame by reducing the PHRR, THR, FIGRA and EHC, and increasing the TTI, which is ascribed by the presence of more non-flammable elements (P, N and C) in the FR composite and also the presence of nitrogen in the FR system that can lead to a nitrogen–phosphorous synergy [20].

It was found that smoke release enhanced with the introduction of P-N-C FR and GnP, as reported in another study in which different types of FRs increased the smoke release of flax fibre composites [20]. Organic P-N flame retardants increase the smoke production, presumably through effective fire resistive reactions in the gas phase, resulting in incomplete combustion during burning [20]. However, the CO_2_ yield, i.e., the combustion efficiency, decreased in the FR treated fibre composite by 12% (Table 3) similar to the other research study where hemp fibre epoxy composites showed lower CO_2_ yield after the fibre silica treatment [19]. This was also found in the investigation carried out by Szolnoki et al. [21] where hemp fabrics were treated in phosphoric acid solution and amine-type silane solution to provide two different fibre treatments prior to making the treated hemp fabric epoxy composites. In their study, the CO_2_ yield decreased upon the incorporation of the treated fabrics as a reinforcement material relative to that of the untreated hemp fabric composite similar to this work. Moreover, it was also detected that the CO yield increased for both types of treated hemp fibre epoxy composites and the mean specific extinction area (SEA), i.e., soot yield, enhanced for silane treated fibre composite as compared to that of the untreated fabric counterpart. This was similar for the FR treated fibre composite for which both CO yield and SEA increased significantly. The GnP composite showed less influence on the composite efficiency with only a small increase in the CO yield and SEA. The FTIR apparatus was calibrated to quantitatively measure the following gases: carbon dioxide (CO_2_), carbon monoxide (CO), hydrogen cyanide (HCN), hydrogen chloride (HCl), hydrogen fluoride (HF), hydrogen bromide (HBr), sulfur dioxide (SO_2_), nitrogen dioxide (NO_2_) and nitric oxide (NO). The FTIR analysis only showed quantifiable levels of CO_2_ and CO in the tests. The production of these gases is given in Table 3 as mass-loss yields during the flaming periods of the tests. Nitric oxide (NO) was detected (limit of detection (LOD) = 4 ppm) for all composites during the time of the most intense burning but the concentration levels hovered around the limit of quantification (LOQ = 3 × LOD). However, the highest peak concentration was detected from the FR composite (~16 ppm). None of the remaining calibrated gases were detected in the tests. Data on CO_2_ and CO was also available from the cone calorimeter instrumentation. The agreement with the FTIR results was good for CO_2_, but more variable for CO. As the FTIR was calibrated for CO in the low concentration range (below ~100 ppm) relevant here, it was decided to use the data from the FTIR.

### 3.2. Dynamic Mechanical Analysis

**Figure 8:**
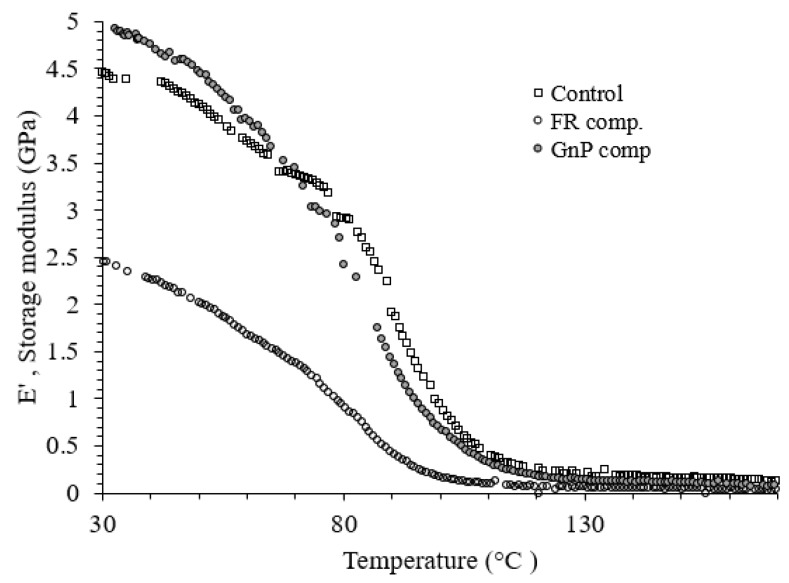
Dynamic mechanical analysis (storage modulus vs. temperature) of the composites.

**Figure 9:**
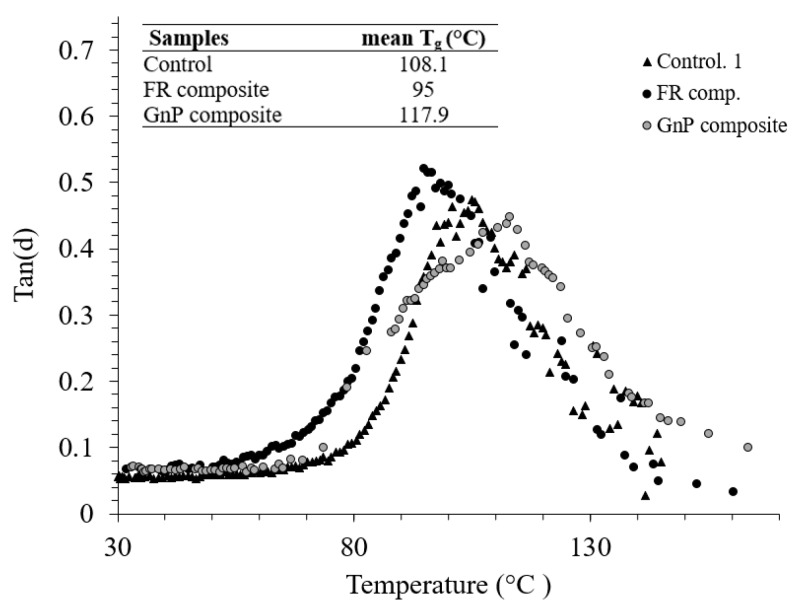
Loss factor (Tan δ) vs. temperature from the dynamic mechanical analysis (DMA) tests.

DMA tests were performed to evaluate the effect of fillers on the viscoelastic behaviours of jute fabric Elium^®^ composites. The recorded storage moduli (E’) of the Control, FR composite and GnP composites at 30 °C were 4.47, 2.45 and 4.90 GPa, respectively, as displayed in Figure 8 and Figure 9. At glassy phase region (below 70 °C), GnP composite showed higher E’ than other composites, indicating the capability of the composite to elastically store more energy [22]. With the increment in temperature, a fast, continuous reduction in storage modulus obtaine and an abrupt reduction in E΄ started at around 80 °C, correlated to the glass transition zone.

As shown from the peak of loss factor (Figure 9) the glass transition temperature (T_g_) was recorded 108 °C, 95 °C and 117 °C for the Control, FR composite and GnP composite, respectively. As compared to natural fibre epoxy composite with and without the incorporation of FRs as seen in the open literature [22,23], the Control and FR Elium^®^ composites demonstrated higher T_g_. Besides, T_g_ decreased after addition of FR additive to the composite system for both the natural fibre epoxy, as demonstrated in the mentioned studies, and Elium^®^ composites. However, nanofillers, i.e., GnP particles constrained the mobility level of micro-molecules of Elium^®^ matrix segments, which explains the improvement in T_g_ [24,25].

### 3.3. Morphological Structure of the Composites

**Figure 10:**
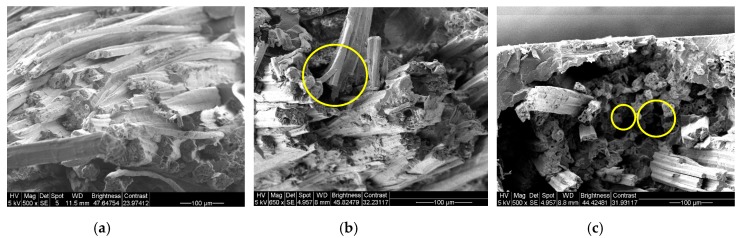
SEM micrographs of fracture surface of (**a**) the Control, (**b**) GnP composite and (**c**) FR composite after the tensile test at 500× magnification.

The SEM images of the fracture surface of the composites are shown in Figure 10. Figure 10a demonstrates good interfacial interaction between the Elium^®^ matrix and fibre for the Control sample. The fibre breakage was observed in the micrograph, the fibres were covered in the Elium^®^ matrix and no indication of delamination was seen. For the GnP composite, some fibre pull-out was seen (Figure 10b) whereas the fibre pull-out locations were obvious in the FR composite (Figure 10c) which are indicated in yellow circles. As observed in the FR composite sample, the fibre-matrix adhesion is poor which results in fibre debonding, attributed to the incompatibility of FR and Elium^®^ matrix.

### 3.4. Mechanical Performance

**Figure 11:**
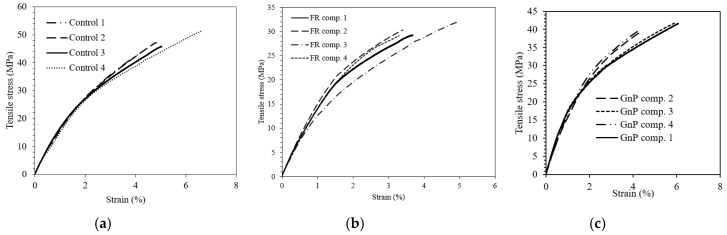
The tensile stress-strain curves of (**a**) Control, (**b**) FR composite and (**c**) GnP composite.

The tensile behaviours of the Control, GnP and FR composite laminates are displayed in Figure 11 and Table 4. The tensile strength of composite laminates was 46.7, 40.4 and 30 MPa, and the moduli were 8.2, 7.9 and 6.9 GPa for the Control, GnP composite and FR composite, respectively. This demonstrates a reduction in tensile properties after grafting the fillers onto the fabrics. The decrease in the tensile properties of GnP composite could be due to a decrease in wettability between fibre and polymer matrix after the inclusion of fillers. Therefore, at some zones the fibres could be more prone to be pulled out from the matrix rather than breaking (fibre breakage). As the tensile test is mainly fibre dependent, this affects the tensile performance more than bending test which is more matrix dependent. The tensile performance drop was more pronounced for the FR composite as it had higher amounts of additive in the composite. Furthermore, as compared to GnP composite, FR composite was found to experience an obvious reduction in the tensile propertie; this phenomenon could be explained by the fact that FR is a chemical composition and GnP is a nanomaterial. Hence, larger particle size can act as a nucleating agent, which results in decrease of mechanical properties. Basically, these additives act as impurities and reduce the adhesion between fibres and polymer matrix [24,26], which was demonstrated in the SEM micrographs. This negatively affects the load transfer from the polymer matrix to the fibres. Szolnoki et al. [21] reported that the so-called thermotex treatment of woven hemp fabrics using phosphoric acid solution that produced 1.7 mass percent of P content and the hemp fabric treatment using amine-type silane which added 4.9 mass percent of Si onto the fabric reduced the tensile strength and modulus of 30 volume percent woven hemp fabric epoxy composite. 

**Figure 12:**
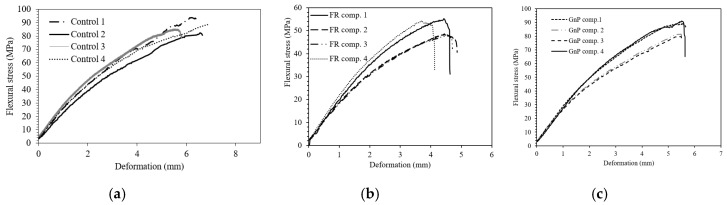
The bending stress-displacement curves of (**a**) Control, (**b**) FR composite and (**c**) GnP composite.

The flexural behaviour of the Control, GnP and FR composites were studied, and the results are shown in Figure 12 and Table 5. The flexural strength and modulus were 87.13 MPa and 4.29 GPa for the Control composite whereas the addition of FR fillers into the composite reduced these parameters. The reduction in bending properties were more significant for FR composite as more incompatible particles were present in the polymer composite. The addition of the fillers into the composites led to premature failure at the interface of fibre and Elium^®^ matrix. The inclusion of GnP filler had little impact on the flexural performance of the composite.

## 4. Conclusions

The jute fabrics were treated with an inexpensive, bio-degradable, scalable, new, simple aqueous organic phosphonate ester FR coating and GnP additives containing C element. GnP and organic phosphonate ester based FRs promoted a char formation to protect the bulk of substrate on combustion, which led to the reduced PHRR by 8% and 25%, respectively, whereas the TSR increased. With the inclusion of FR filler in the composite, the CO yield enhanced, and the CO_2_ yield dropped. However, the incorporation of GnP additives into the composite did not affect the CO_2_ and CO yield. For the DMA results, it was found that GnP composite had an enhanced Tg (i.e., 117.9 °C) relative to that of the Control. As expected, the addition of the fillers was observed to decrease the interfacial bonding between the Elium^®^ matrix and the fibres as demonstrated by the SEM micrographs, which resulted in the reduced mechanical performances. In this study, the flame resistive fillers in the form of solution were used to treat the fabrics to evaluate their respective composite performance; in the future work, the infusible fire retardant mats will be used to protect the top and bottom layers of composite laminates to provide enhancement in fire retardancy, as the PMMA is highly flammable and is exposed to the heat on the surface of the composite in the first place. Also, these mats could potentially improve or at least maintain the mechanical properties of natural fibre composites, unlike treated fabric composites which normally result in the reduction of mechanical properties. 

## Figures and Tables

**Table 1:** Sample designation.

Sample	GnP (wt.%)	FR		Fibre Volume Fraction (%)
		Carbon (wt.%)	Phosphorous (wt.%)	
Control	-	-	-	40
FR composite	-	0.22	0.72	40
GnP composite	1	-	-	40

**Table 2:** Results from the Cone calorimeter tests. The data refers to the flaming phase of the tests.

Sample	TTI (s)	t_PHRR_ (s)	PHRR (kW/m²)	FIGRA (kW/m²·s)	THR (MJ/m^2^)	EHC (MJ/kg)	TSR (m^2^/m^2^)	SEA (m^2^/kg)
Control	21	64	552	8.6	33.5	20.6	148	90.3
FR composite	28	56	410	7.3	35.3	19.1	241	131.0
GnP composite	24	60	510	8.5	40.0	20.9	188	97.9

TTI: time to ignition, t_PHRR_: time to PHRR, PHRR: peak heat release rate, FIGRA: the fire growth index, THR: total heat release, EHC: the effective heat of combustion, TSR: total smoke release, SEA: specific extinction area.

**Table 3:** The yields of gases evolved from the samples as determined by the FTIR. The data refers to the flaming phase of the tests.

Sample	CO_2_ (g/g)	CO (g/g)
Control	1.94	0.014
FR composite	1.71	0.062
GnP composite	1.94	0.016

**Table 4:** Tensile properties of the composites.

Samples	Tensile Strength (MPa)	Tensile Modulus (GPa)	STD (Strength)	STD (Modulus)
Control	46.7	8.2	3.0	0.5
GnP composite	40.4	7.9	1.2	0.2
FR composite	30	6.9	1.7	0.4

**Table 5:** Flexural properties of the composites.

Composites	Flexural Strength (MPa)	Flexural Modulus (GPa)	STD (Strength)	STD (Modulus)
Control	87.13	4.29	4.43	0.06
GnP composite	84.13	4.06	5.98	0.16
FR composite	51.34	2.79	3.27	0.25
